# Biosensor-Based Comparison of Stress Responses in Qingtian Paddy Field Carp (*Cyprinus carpio* var. *qingtianensis*) and Xingguo Red Carp (*Cyprinus carpio* var. *singuonensis*) Under Acute Shallow Water Conditions

**DOI:** 10.3390/biology14091303

**Published:** 2025-09-20

**Authors:** Tengyu Liu, Rui Han, Yuhan Jiang, Jiamin Sun, Haiyun Wu, Qigen Liu

**Affiliations:** 1 Research Centre of the Ministry of Agriculture and Rural Affairs on Environmental Ecology and Fish Nutrition, Shanghai Ocean University, Shanghai 201306, China; liutengyu007@gmail.com (T.L.); 18861838941@163.com (R.H.); lily1993on@163.com (Y.J.); jmsun@shou.edu.cn (J.S.); 2 Department of Ocean Sciences, Tokyo University of Marine Science and Technology, 4-5-7 Konan, Minato-ku, Tokyo 108-8477, Japan; 3 Key Laboratory of Exploration and Utilization of Aquatic Genetic Resources, Ministry of Education, Shanghai Ocean University, Shanghai 201306, China; 4 Shanghai Engineering Research Center of Aquaculture, Shanghai Ocean University, Shanghai 201306, China

**Keywords:** glucose biosensor, Qingtian paddy field carp, Xingguo red carp, stress response, real-time monitoring

## Abstract

The domestication of common carp in rice paddies (5–20 cm depth) is challenging, as the fish must withstand drastic fluctuations in temperature and dissolved oxygen, restricted movement, and bird predation without the option of diving. The effects of stress responses in different species of carp in shallow-water environments remain poorly understood, particularly with fluctuating water levels where real-time monitoring is challenging. This study employed a glucose biosensor system enabling real-time monitoring, together with biochemical analysis techniques capable of evaluating multiple physiological indicators, to investigate shallow-water adaptation in Qingtian paddy field carp and Xingguo red carp. Our results quantitatively reveal, for the first time, the differing physiological stress thresholds of the two carp strains under shallow water. The Qingtian paddy field carp exhibited a higher tolerance to shallow water and showed faster recovery from prolonged stress. Furthermore, the total cholesterol and triglyceride contents of Qingtian paddy field carp gradually increased with prolonged shallow-water stress, reflecting the activation of lipid metabolic pathways. These findings highlight the advantages of biosensor technology in aquatic stress research and a strong support of the core element of paddy domesticated carp in the Globally Important Agricultural Heritage Systems.

## Simple Summary

Water depth is a crucial environmental factor influencing temperature, oxygen availability, light intensity, and ultimately the physiology, behavior, and distribution of aquatic animals. This study investigated the physiological responses of two common carp varieties—the Qingtian paddy field carp, a core component of the Qingtian rice-fish co-culture system, and the Xingguo red carp, a typical pond-domesticated species—to extreme shallow aquatic environments (5–20 cm depth). Since traditional blood sampling fails to capture dynamic stress levels, we used a real-time glucose biosensor combined with biochemical assays to continuously monitor stress and adaptive reactions. The results show that the Qingtian carp exhibits superior adaptation to shallow water conditions, marked by higher stress tolerance, faster recovery, and efficient lipid-based energy utilization. These findings highlight the value of biosensor-based approaches in aquaculture and affirm the critical role of the Qingtian carp in sustainable integrated rice–fish systems.

## 1. Introduction

Domestication is an evolutionary process involving artificial selection, through which animals undergo significant changes in their behavior, physiology, and external traits. This process took place independently across multiple regions and had a profound influence on human societies, marking the onset of the Neolithic transition. It is characterized not only by its gradual progression but also by its substantial techno-economic impact, shifting human subsistence from hunting and gathering to systematic food production [1,2]. The common carp (*Cyprinus carpio*) is considered one of the most economically important fish species globally, ranking fourth in overall aquaculture production, and has the longest history of domestication among aquaculture species [3,4,5]. Over centuries, it has been domesticated in diverse environments, such as ponds and rice paddies, for various purposes including consumption and ornamental display [6]. In ponds, domestication occurs under relatively stable conditions with deeper water, reduced diurnal fluctuations in temperature and dissolved oxygen, and the presence of aquatic plants that buffer water quality and provide shelter. Such environments closely mimic the natural habitats of carp in lakes and rivers, minimizing external stress and promoting predictable growth. In contrast, paddy fields expose carp to shallow water levels, restricted water exchange, and pronounced diurnal fluctuations in temperature and dissolved oxygen [7,8]. For example, during summer 2022 in Qingtian, Zhejiang Province, China, water temperatures in rice paddies ranged between 31.3 and 40.2 °C at depths of 5–22 cm, whereas adjacent ponds showed smaller diurnal variations (3–5 °C) and greater water depths (1–2 m) [9,10]. Such contrasting domestication environments have shaped distinct adaptive traits in different carp lineages. Xingguo red carp (*Cyprinus carpio var. singuonensis*), a nationally protected non-exported Chinese carp breed, is a classic pond-domesticated species prized for hybrid vigor and ornamental value. Renowned for its stable economic traits and palatability, it holds strategic importance in freshwater fish germplasm conservation and genetic breeding [11,12]. Conversely, the Qingtian paddy field carp (*Cyprinus carpio var. qingtianensis*) represents the most prominent lineage domesticated under paddy field conditions. As a fundamental component of the Qingtian rice–fish co-culture system, which was recognized by the FAO in 2005 as one of the first pilot sites of the Globally Important Agricultural Heritage Systems (GIAHS), this strain has demonstrated exceptional tolerance to shallow water, elevated temperatures, and hypoxia. In this integrated system, carp interact with rice plants by loosening soil and promoting nutrient cycling, while controlling weeds and pests, thereby reducing the need for chemical fertilizers and pesticides. Consequently, the rice–fish co-culture system enhances rice yield and quality, provides carp as an additional food source, generates economic benefits for farmers, and contributes to biodiversity conservation and sustainable agriculture [13,14,15,16].

Different domestication methods will adapt the carp to different stressful environments in order to restore and maintain internal balance [17]. This adaptation process is closely linked to the physiological stress response, which is characterized by the activation of the neuroendocrine system, resulting in a cascade of metabolic and physiological changes. Indeed, exposure to stress rapidly activates the hypothalamic–pituitary–interrenal (HPI) axis, leading to the release of neuromediators and stress hormones such as adrenaline, noradrenaline, and cortisol [18,19]. The effects of these molecules are structural, hydromineral, hematological, and metabolic. In particular, they stimulate metabolic pathways that elevate plasma glucose levels, thereby supplying additional energy to help the organism cope with stress [20]. Furthermore, different domestication methods have driven carp strains to evolve specialized molecular and metabolic regulation. In the Qingtian paddy field carp, for example, this is manifested by the upregulation of endoplasmic-reticulum-associated genes, such as HSPs, and the AMP-activated protein kinase (AMPK) signaling pathway—which is essential for protecting these fish against the high temperatures and hypoxia typical of rice paddies [21,22]. These carp also cope with dramatic diurnal oxygen fluctuations by boosting gluconeogenic energy supply via glutamate–glutamine metabolism, while simultaneously restoring metabolic balance through the depletion of accumulated glutamate/glutamine, the suppression of glycerophospholipid synthesis, and the promotion of purine recycling [23,24].

Water depth is an important environmental factor, which not only determines the vertical profiles of water temperature, oxygen, light intensity, etc., but also affects the physiology, behavior, sense of safety, and distribution of aquatic animals; e.g., fish in shallow rice paddies can only move around horizontally; they cannot dive to deep water when facing predation by a bird and thus may be more stressed when kept in shallow water such as in the rice paddies. Dynamic environmental stressors, such as rapid fluctuations in water levels, present challenges for aquatic organisms. However, monitoring their physiological responses in real time remains difficult. Blood sampling makes it difficult to capture the transient stress state of fish. There is still a gap in research on the physiological mechanisms of Qingtian paddy field carp adapting to shallow-water extreme habitats, specifically, a lack of systematic analysis of the response mechanisms to shallow-water stress, not to mention the comparative study on the adaptive potentials of pond-raised *Cyprinus carpio* in shallow-water environments of rice paddies. Here, we address this issue by using implantable biosensor technology to enable continuous glucose monitoring in freely moving fish. Given the long-term domestication of Qingtian paddy field carp under shallow, thermally fluctuating, and hypoxic paddy environments, we hypothesized that this variety has developed superior physiological tolerance to acute shallow-water stress compared to pond common carp. We hypothesized that carps under different domestication conditions (rice paddy vs. fish pond) may display different stressful responses to shallow water. To explore the above scientific problems, this study simulated the shallow-water habitat of the paddy field by constructing a gradient water level control system, used biosensor technology to monitor the real-time blood glucose concentration of Qingtian paddy field carp and the control species Xingguo red carp, and carried out simultaneous quantitative analyses of physiological indices in the hepatopancreas, gill tissues, and serum. The results of this study will provide a theoretical basis and practical guidance for increasing the diversity and sustainability of paddy field ecosystems.

## 2. Materials and Methods

### 2.1. Reagents

Glucose oxidase (GOx; from *Aspergillus niger*; 137,100-unit g^−1^) was obtained from Sigma (St. Louis, MO, USA). Bovine serum albumin (BSA), heparin sodium, glutaraldehyde (grade I, 25% aqueous solution), and 5% Nafion^®^ dispersion solution were purchased from Aladdin (Shanghai, China). Phosphate-buffer solution (PBS, 0.1 M, pH 7.8) was purchased from Codow (Guangzhou, China).

### 2.2. Test Environment

The Qingtian paddy field carp used in this experiment were procured from Yugong Ecological Agricultural Technology Co., Ltd. (Qingtian, Lishui, China), and the Xingguo red carp were purchased from Xingguo County, Jiangxi Province, China. Healthy and active individuals were selected based on the absence of visible lesions, malformations, or external parasites, intact fins and scales, normal body coloration, and vigorous swimming behavior. To minimize size-related variation, individuals with similar body sizes were chosen, with an average body length of 25.45 ± 1.5 cm, a body height of 10.04 ± 1.3 cm, and a body weight of 206 ± 5.03 g. After transportation, they had a 7-day acclimation period at the laboratory of Shanghai Ocean University. A total of 25 Qingtian paddy field carp and 25 Xingguo red carp were randomly assigned to four 200 L experimental tanks (water depth: 50 cm) for acclimation. Throughout the entire experimental period, including both the acclimation and formal experiment phases, the water quality was strictly controlled and remained consistent across all treatment groups. The environmental parameters were maintained as follows: half of the water in each tank was replaced at 9:00 and 18:00 daily with aerated water to ensure water quality; the water temperature was kept at 26 ± 0.5 °C; the dissolved oxygen concentration was maintained above 6.5 mg·L^−1^; and the pH level was stable within the range of 7–8. Additionally, key water quality parameters were monitored twice daily using a multiparameter water quality meter (YSI ProDSS, Yellow Springs, OH, USA), and the use of an air pump (LX-813, 65 W, 65 L/min, Xiaolongju, Hangzhou, China) maintained the dissolved oxygen concentration within safe ranges. Fish were fed once daily at 8:00 with a commercial carp diet containing 30% crude protein and 3% crude lipid. Feeding was adjusted to apparent satiation to avoid overfeeding. Feeding was suspended one day prior to the start of the formal experiment to minimize postprandial metabolic effects and prevent fecal residues from influencing water quality during stress trials. This research was authorized by Shanghai Ocean University’s Institutional Animal Care and Use Committee (IACUS), Shanghai, China (Approval Code: SHOW-DW-2023-025).

### 2.3. Enzyme Sensor Preparation

A 15.0 mm Teflon-coated Pt-Ir wire was used as the working electrode in the biosensors’ preparation, while the counter/reference electrode consisted of Ag/AgCl paste. The working electrode with the Teflon layer removed is sequentially immersed in 5% Nafion^®^ solution and a mixed GOx and BSA solution. To facilitate cross-linking between GOx and BSA, the sensor was placed in a Petri dish containing glutaraldehyde for three hours at a constant temperature of 35 °C. The sensors were stored in PBS at 4 °C before use.

### 2.4. Sensor Performance Evaluation in Buffer Solution

The sensor was connected to a potentiostat (+650 mV) for amperometric glucose measurement and immersed in 15 mL of PBS. After the output current value became stable, a standard glucose solution (5000 mg dL^−1^) was added to the PBS to achieve a glucose concentration of 5, 10, 15, 20, 25, 30, 40, 50, 70, 90, 120, or 150 mg dL^−1^, and the output current value was recorded. A calibration curve was obtained by plotting the current response against glucose concentration, and linear regression analysis was used to evaluate their correlation. The correlation coefficient (R) was calculated to assess the linearity of the sensor performance.

### 2.5. Sensor Implantation

Sensor implantation and fixation were carried out as follows. The test fish was initially captured using a net and then immersed in an anesthetic solution (MS-222). A 23-G catheter consisting of an outer Teflon layer and an inner puncture needle was inserted into the abdominal interstitial fluid (AISF) for sensor implantation. The biomedical glue, which had ethyl cyanoacrylate monomer as the main ingredient, was used to secure the sensor in place. In order to minimize the effect of the sensor implantation operation on the fish, it is necessary to allow the fish to recover for more than 12 h after completing the operation.

### 2.6. Real-Time Monitoring of the Fish Stress Response Using the Biosensor System

**Figure 1:**
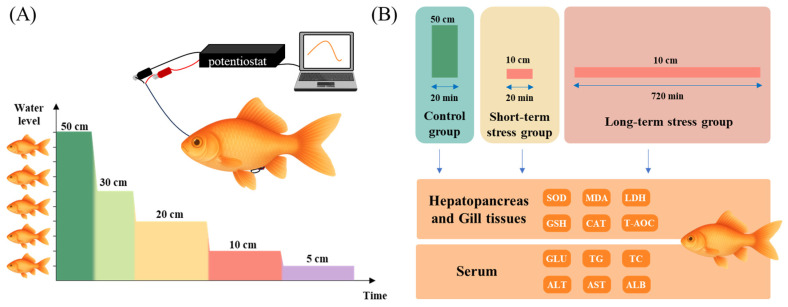
Experimental design for comparing stress response between Qingtian paddy field carp and Xingguo red carp under acute shallow water conditions. (**A**) The glucose sensor, the potentiostat, and the computer were used to record the changes in glucose concentration after the fish were subjected to shallow-water stress in real time. (**B**) The control group was maintained in water with a depth of 5× body height (50 cm) for 20 min, while the short-term and long-term stress groups were exposed to a shallow water depth of 1× body height (10 cm) for 20 min and 720 min, respectively. After the treatments, samples were collected from the hepatopancreas, gill tissues, and serum to analyze physiological indices.

The experimental fish recovered overnight, and the experiment was initiated only after the output current had stabilized, defined as fluctuations within ±2% of the mean over a continuous 30 min period and a maximum current not exceeding 200 nA. Figure 1A shows the real-time water level stress experiment on Qingtian paddy field carp and Xingguo red carp. The initial water level was set at 5× body height (50 cm), and the experimental water levels were set at 3× body height (30 cm), 2× body height (20 cm), 1× body height (10 cm), and 0.5× body height (5 cm). At the beginning of the experiment, we first stayed at the initial water level for 10 min to ensure the output current was stable, then passed the siphon tube, rapidly lowered the water level to 3× body height (30 cm), and recorded the current changes for 20 min. Then, we continued to lower the water level to each experimental depth through the siphon tube and measured the change in the output current value for 20 min at each water level (Figure 1A). Apart from the intentionally manipulated water level, all other conditions were kept constant, and no significant differences were observed among the experimental groups. At the end of the experiment, the experimental fish were quickly moved to the anesthetic solution, and the glucose sensor was removed after the fish were anesthetized. A total of 1 mL of blood was drawn through the caudal vasculature, the blood was centrifuged, and the supernatant was taken and stored in a −20 °C refrigerator. Each group included three parallel replicates (n = 3), resulting in 6 experimental fish samples.

### 2.7. Water Level Stress Experimental Design and Sample Collection

Figure 1B shows the water level stress experiment on Qingtian paddy field carp and Xingguo red carp. For each species, three experimental groups were established: a 5× body height (50 cm) control group, a 1× body height (10 cm) short-term stress group (20 min), and a 1× body height (10 cm) long-term stress group (6 h). Each group included four parallel replicates (n = 4), producing 24 experimental fish samples. Before the experiment, the fish underwent a 7-day acclimation period, and feeding was halted one day before the formal experiment. For the stress groups, the water level was rapidly lowered to 1× body height using the siphon tube and maintained at this level for either 20 min or 6 h, depending on the group. All conditions other than water level remained stable throughout the experiment and showed no significant variation across the experimental groups.

After the stress period ended, four fish were sequentially taken from each experimental tank and immersed in an anesthetic solution (MS-222). The fish’s body surface was cleaned with alcohol, and 1 mL of blood was collected from the caudal vasculature. The blood samples were transferred into 2 mL anticoagulant centrifuge tubes and kept at 4 °C. Following blood collection, the fish were dissected to extract the hepatopancreas and gills. These tissues were rinsed with physiological saline to remove surface blood, then blotted dry with filter paper. The hepatopancreas and gills from each fish were placed into separate 2 mL cryogenic vials, pre-cooled with liquid nitrogen, and promptly stored at −80 °C. The collected blood samples were placed in a refrigerator at 4 °C for 24 h and then centrifuged at low temperature (4 °C, 3000 r/min, 10 min). The supernatant was then extracted using a pipette and stored at −80 °C for subsequent biochemical analyses.

### 2.8. Data Analysis of Biosensor

The real-time glucose monitoring biosensor system collects data at a frequency of one reading per second. However, these measurements may be affected by transient noise caused by fish movement. To ensure the reliability of the data used for analysis, outlier detection and removal were performed using two widely adopted statistical methods: the Z-score method and the Interquartile Range (IQR) method. Subsequently, the cleaned data were resampled using a sliding window approach with a window size of 60 data points and a step size of 1. These parameters were selected to balance temporal resolution with data stability and to ensure sufficient sensitivity for detecting short-term behavioral changes. The glucose biosensor was calibrated using the single-point calibration method, and the converted glucose concentrations are displayed chronologically [25,26].

### 2.9. Biochemical Analysis

In this study, biochemical parameters in the hepatopancreas and gills were measured using commercially available assay kits (Nanjing Jiancheng Bioengineering Institute, China). For each parameter, four biological replicates were included per experimental group. Commercial kits were used for the detection of biochemical indicators, which included superoxide dismutase (SOD), malondialdehyde (MDA), lactate dehydrogenase (LDH), catalase (CAT), reduced glutathione (GSH), total antioxidant capacity (T-AOC), and total protein (TP). Blood levels of glucose (GLU), albumin (ALB), alanine aminotransferase (ALT), aspartate aminotransferase (AST), total protein (TP), triglyceride (TG), and total cholesterol (TC) were measured by BS-450 (Shenzhen Mairui Biomedical Electronics Co., Ltd., Shenzhen, China). The specifications of the commercially available assay kits used for biochemical measurements are summarized in Table A1.

### 2.10. Statistical Analysis

Statistical analyses were conducted using SPSS 26.0 (SPSS Inc., Chicago, IL, USA). Prior to one-way analysis of variance (ANOVA), data were tested for normality using the Shapiro–Wilk test and for homogeneity of variance using Levene’s test. When the assumptions were met, ANOVA was used to evaluate the main effects of water level stress. Duncan’s multiple range test was applied for post hoc comparisons, with statistical significance set at *p* < 0.05. All values were expressed as mean ± standard error of the mean (SEM).

## 3. Results

### 3.1. Biosensor Calibration Curve

**Figure 2:**
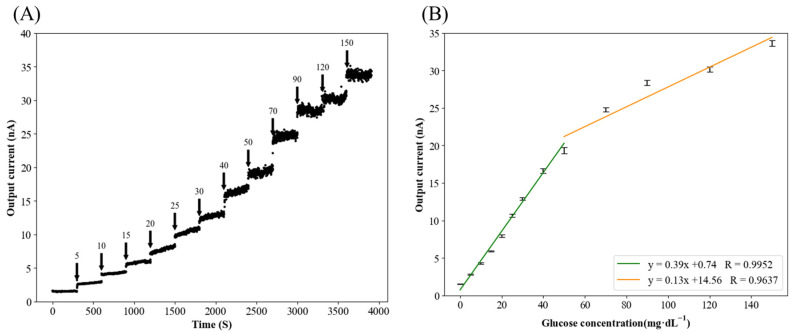
The response curve and calibration curve of the glucose biosensor. (**A**) Response curve of the sensor in the PBS solution (0.1 M, pH 7.8). The number above the arrow in the figure is the glucose concentration in the PBS solution after adding the glucose standard solution. (**B**) The calibration curve of the sensor is according to the data in Figure 2A.

Figure 2A shows the response data of the sensor’s output current value upon incremental addition of glucose solution. The arrows indicate the final glucose concentrations in the solution after each standard addition. Figure 2B presents the calibration curve of the sensor. The sensor’s output current value significantly correlated with the glucose concentration (0–50 mg dL^−1^: y  =  0.39x + 0.74, R  =  0.9952; 50–150 mg dL^−1^: y  =  0.13x + 14.56, R  =  0.9637) in the PBS from 0 to 150 mg dL^−1^.

### 3.2. Real-Time Stress Response Monitoring of Qingtian Paddy Field Carp and Xingguo Red Carp

**Figure 3:**
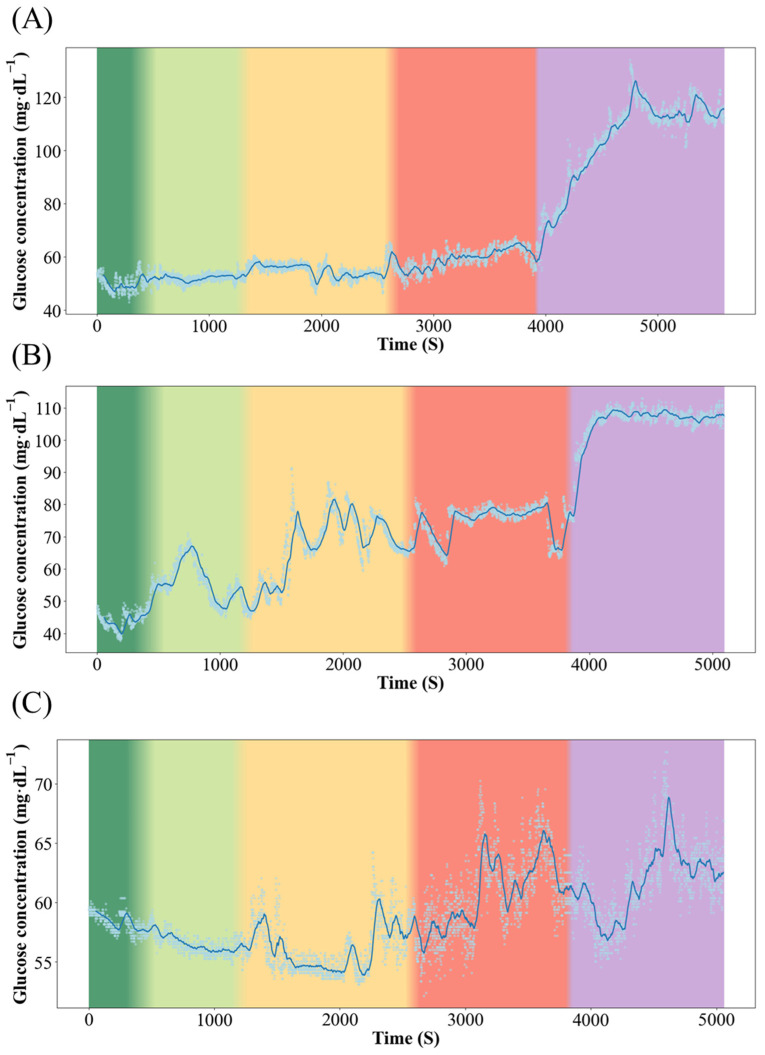
Dynamics of blood glucose concentration in the Qingtian paddy field carp. Color changes from left to right represent changes in water level, where dark green represents 5× body height (50 cm); light green represents 3× body height (30 cm); yellow represents 2× body height (20 cm); red represents 1× body height (10 cm); and purple represents 0.5× body height (5 cm). (**A**–**C**) represent three individual replicates of Qingtian paddy field carp subjected to the same experimental conditions (n = 3).

**Figure 4:**
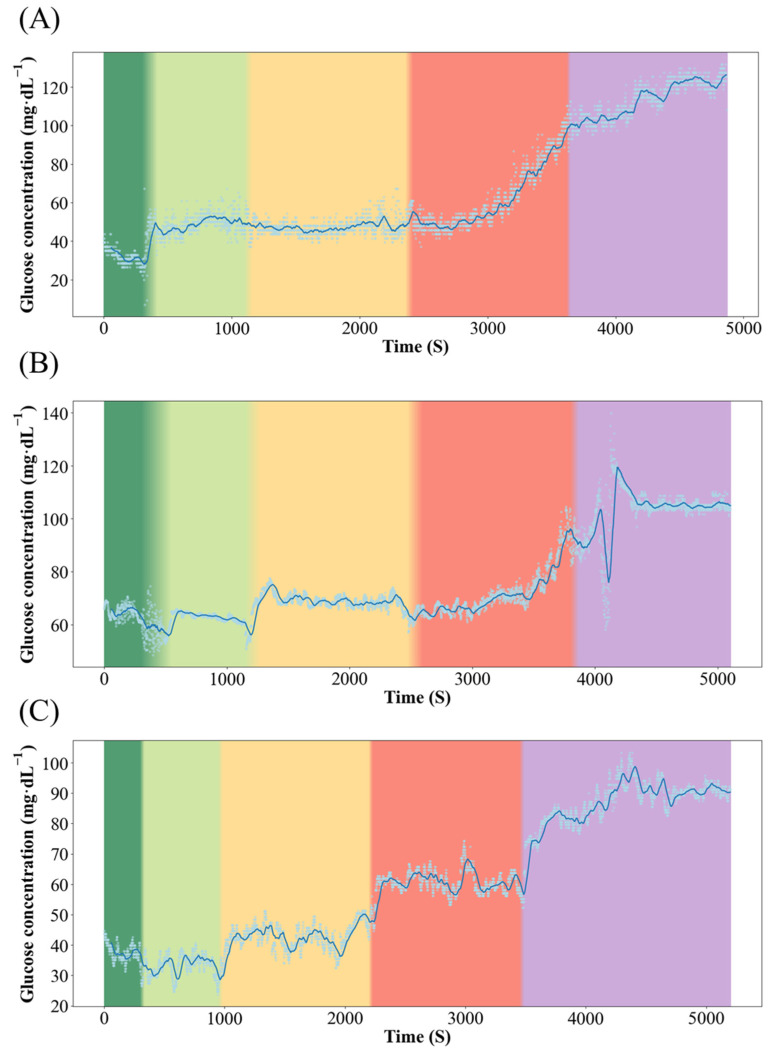
Dynamics of blood glucose concentration in the Xingguo red carp. Color changes from left to right represent changes in water level, where dark green represents 5× body height (50 cm); light green represents 3× body height (30 cm); yellow represents 2× body height (20 cm); red represents 1× body height (10 cm); and purple represents 0.5× body height (5 cm). (**A**–**C**) represent three individual replicates of Xingguo red carp subjected to the same experimental conditions (n = 3).

Figure 3 and Figure 4 show the dynamics of blood glucose concentration in Qingtian paddy field carp and Xingguo red carp, respectively. In Xingguo carp, the glucose concentration rose sharply already at 1× body height (red module, 10 cm), whereas in Qingtian paddy field carp, a significant glucose increase was observed only when the water depth decreased to 0.5× body height (purple module, 5 cm). These results indicate that Qingtian paddy field carp possess a stronger tolerance to shallow-water stress.

### 3.3. Physiological Indices of Hepatopancreas in Qingtian Paddy Field Carp and Xingguo Red Carp Under Acute Shallow-Water Stress

**Figure 5:**
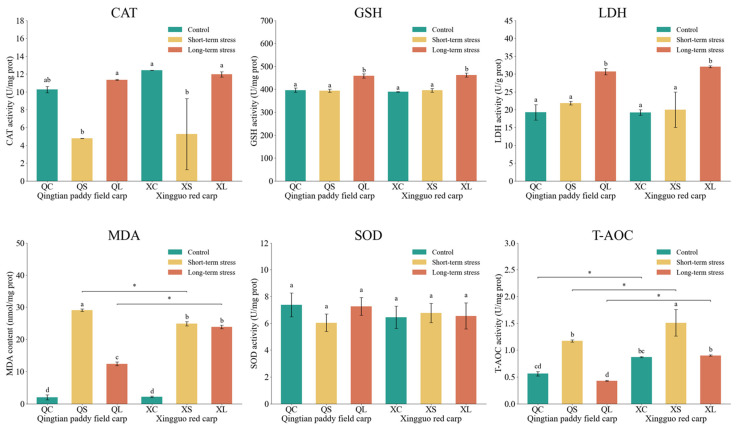
Effects of acute shallow-water stress on physiological indices in the hepatopancreas of Qingtian paddy field carp and Xingguo red carp. QC represents the Qingtian paddy field carp control group, QS represents the short-term stress group, and QL represents the long-term stress group. XC represents the Xingguo red carp control group, XS represents the short-term stress group, and XL represents the long-term stress group. Values are means (n = 4), with their standard error of the mean (SEM) represented by vertical bars. Bars bearing the same letters were not significantly different (*p* > 0.05). The asterisk (*) indicates a significant difference between two species under the same conditions (* *p* < 0.05).

The effects of acute shallow-water stress on physiological indices in the hepatopancreas of Qingtian paddy field carp and Xingguo red carp are shown in Figure 5. Following exposure to acute shallow-water stress, MDA concentrations in the hepatopancreas of both Qingtian paddy field carp and Xingguo red carp increased significantly in the short-term stress groups compared to their respective controls (*p* < 0.05), consistent with the overall oxidative stress response. In Qingtian paddy field carp, MDA levels in the long-term stress group were significantly lower than those in the short-term stress group (*p* < 0.05), whereas no significant difference was observed in Xingguo red carp (*p* > 0.05). CAT activity was significantly reduced in the short-term stress groups of both species relative to the controls (*p* < 0.05), while the CAT activities essentially returned to their original levels after long-term stress. No significant differences in SOD activity were detected among the control, short-term, and long-term stress groups in either species (*p* > 0.05). LDH and GSH activities in both species showed no significant changes in the short-term stress groups compared to the control groups (*p* > 0.05), but both were significantly increased in the long-term stress groups (*p* < 0.05). T-AOC activity in both species was significantly higher in the short-term stress groups than in the control groups (*p* < 0.05), with no significant difference observed in the long-term stress groups (*p* > 0.05).

### 3.4. Physiological Indices of Gill Tissues in Qingtian Paddy Field Carp and Xingguo Red Carp Under Acute Shallow-Water Stress

**Figure 6:**
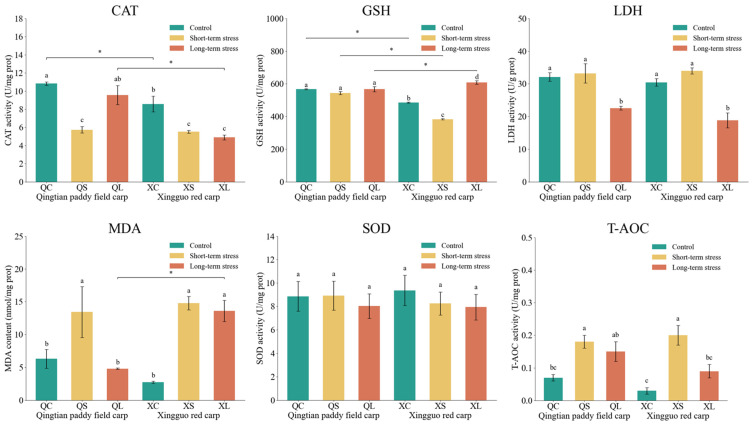
Effects of acute shallow-water stress on physiological indices in gill tissues of Qingtian paddy field carp and Xingguo red carp. QC represents the Qingtian paddy field carp control group, QS represents the short-term stress group, and QL represents the long-term stress group. XC represents the Xingguo red carp control group, XS represents the short-term stress group, and XL represents the long-term stress group. Values are means (n = 4), with their standard error of the mean (SEM) represented by vertical bars. Bars bearing the same letters were not significantly different (*p* > 0.05). The asterisk (*) indicates a significant difference between two species under the same conditions (* *p* < 0.05).

The effects of acute shallow-water stress on physiological indices in the gill tissues of Qingtian paddy field carp and Xingguo red carp are shown in Figure 6. After exposure to acute shallow-water stress, the MDA concentrations in both Qingtian paddy field carp and Xingguo red carp were significantly higher in the short-term stress groups than in the control groups (*p* < 0.05). The MDA concentration in the long-term stress group of Qingtian paddy field carp was significantly lower than that in the short-term stress group (*p* < 0.05), while no significant difference was observed in Xingguo red carp (*p* > 0.05). CAT activity in Qingtian paddy field carp showed no significant differences between the short-term, long-term stress groups, and the control group (*p* > 0.05). The CAT activity of both carp species was significantly lower in the short-term stress group than in the control group (*p* < 0.05), and increased significantly in the long-term stress group compared with the short-term stress group of Qingtian paddy field carp (*p* < 0.05), while no significant change was observed in Xingguo red carp compared with the short-term stress group (*p* > 0.05). SOD activity in both species showed no significant differences between the stress groups and their respective control groups (*p* > 0.05). LDH activity remained unchanged in the short-term stress groups compared to controls (*p* > 0.05) but showed a significant decrease in the long-term stress groups of both species (*p* < 0.05). GSH activity did not significantly change in Qingtian paddy field carp across treatments (*p* > 0.05); however, in Xingguo red carp, a significant increase was observed in the long-term stress group compared to the control (*p* < 0.05). T-AOC activity was significantly elevated in the short-term stress groups of both species compared to their respective controls (*p* < 0.05).

### 3.5. Physiological Indices of Serum in Qingtian Paddy Field Carp and Xingguo Red Carp Under Acute Shallow-Water Stress

**Figure 7:**
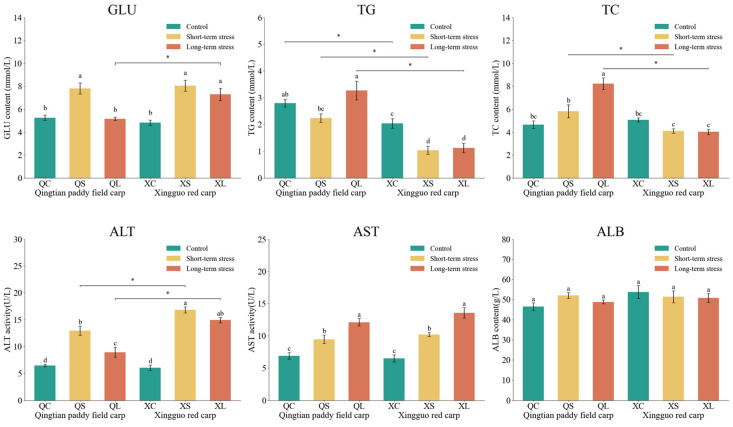
Effects of acute shallow-water stress on physiological indices in Qingtian paddy field carp and Xingguo red carp serum. QC represents the Qingtian paddy field carp control group, QS represents the short-term stress group, and QL represents the long-term stress group. XC represents the Xingguo red carp control group, XS represents the short-term stress group, and XL represents the long-term stress group. Values are means (n = 4), with their standard error of the mean (SEM) represented by vertical bars. Bars bearing the same letters were not significantly different (*p* > 0.05). The asterisk (*) indicates a significant difference between two species under the same conditions (* *p* < 0.05).

The effects of acute shallow-water stress on physiological indices in the serum of Qingtian paddy field carp and Xingguo red carp are shown in Figure 7. In the short-term stress groups, glucose (GLU) levels in both Qingtian paddy field carp and Xingguo red carp were significantly higher than those in their respective control groups (*p* < 0.05). In Qingtian paddy field carp, GLU levels in the long-term stress group were significantly lower than those in the short-term group (*p* < 0.05), while Xingguo red carp showed no significant difference between the short- and long-term groups (*p* > 0.05). No significant changes were observed in triglyceride (TG) levels in Qingtian paddy field carp or in total cholesterol (TC) levels in Xingguo red carp under either stress condition compared to controls (*p* > 0.05). However, TG levels in Xingguo red carp were significantly reduced in both stress groups compared to the control group (*p* < 0.05), and TC levels in Qingtian paddy field carp were significantly elevated in the long-term stress group compared to the control group (*p* < 0.05). Alanine aminotransferase (ALT) activity was significantly increased in the short-term stress groups of both species compared to controls (*p* < 0.05). In Qingtian paddy field carp, ALT activity in the long-term group was significantly reduced compared to the short-term group (*p* < 0.05), whereas no significant difference was observed in Xingguo red carp (*p* > 0.05). Aspartate aminotransferase (AST) activity was significantly elevated in both species under stress conditions relative to controls (*p* < 0.05), while albumin (ALB) levels showed no significant changes across treatment groups in either species (*p* > 0.05).

## 4. Discussion

### 4.1. Biosensors: A New Paradigm for Stress Physiology

The domestication of common carp to shallow water (with a depth between 5 and 20 cm) in rice paddies is a great challenge, since, in such shallow water, they must overcome the great stress caused by drastic variations in water temperature, dissolved oxygen, unsmooth moving, the impossibility of diving to avoid predation by birds, etc. However, due to the lack of appropriate methodology such as the real-time monitoring of physiological stress in dynamic aquatic environments, it has never been evaluated and thus there is a lack of understanding of the adaptation ability and underlying mechanisms in domesticated species or strains. Glucose is a key physiological indicator in this context, as stress-induced hyperglycemia is a hallmark of HPI axis activation in fish. However, conventional blood sampling methods cannot provide continuous, high-resolution monitoring, limiting insights into dynamic stress responses. To overcome this limitation, we employed a minimally invasive, needle-type enzyme biosensor implanted into the abdominal interstitial fluid (AISF) of fish. When a potential of +650 mV was applied, hydrogen peroxide produced during glucose oxidation catalyzed by glucose oxidase (GOx) was electrochemically oxidized at the electrode surface, generating a current proportional to glucose concentration. AISF was chosen because it correlates closely with blood glucose but contains fewer interferents, thereby enabling reliable and prolonged monitoring. Previous studies have also validated glucose biosensors as sensitive and reliable tools for real-time assessment of acute stress in aquatic animals [26,27,28].

The real-time biosensor system was employed for the first time in in vivo experiments on Qingtian paddy field carp and Xingguo red carp, successfully obtaining continuous dynamic data on glucose concentration in response to changes in water depth, thereby confirming that acute shallow-water conditions induce stress in fish. The experiment revealed that in Xingguo red carp, blood glucose concentration increased sharply when the water depth was equivalent to 1× body height, indicating a stress response to water level reduction. In contrast, Qingtian paddy field carp exhibited a significant rise in blood glucose concentration only when the water depth was reduced to 0.5× body height, demonstrating its strong adaptability to shallow-water conditions. This study establishes a novel paradigm for the real-time monitoring of stress physiology in aquatic animals in response to dynamic environmental challenges.

In the acute shallow-water-level stress experiment, Xingguo red carp exhibited significant behavioral stress phenotypes when the water depth reached 1× body height. Characteristic responses included high-frequency jumping behavior and markedly increased respiratory rates. In sharp contrast, Qingtian paddy field carp maintained stable body posture even under extremely shallow water conditions (0.5× body height). These behaviors were recorded by direct observation during the experiment. Notably, such complex behavioral responses cannot be fully captured by a single glucose sensor, highlighting the limitations of the current monitoring system. With the development of artificial intelligence, especially computer vision, new research methods have been developed for exploring the behavior of aquatic animals [29]. In previous studies, an improved YOLOv8 model has been utilized to quantitatively analyze behavioral changes in fish under ammonia-nitrogen stress, offering a new approach for investigating abnormal behaviors in aquatic animals [30]. Additionally, a novel monitoring system was developed that integrates a biosensor for glucose measurement with a triaxial accelerometer sensor for behavioral tracking to assess stress responses in Nile tilapia under acute stress conditions such as ammonia exposure and social interaction [31]. The triaxial acceleration sensor, which record movement along three orthogonal axes (X, Y, Z), can be used to quantify behavioral changes in fish under stress by enabling the calculation of swimming speed and activity levels. Such data would provide high-resolution and continuous measurements of stress-related behaviors, complementing biochemical and physiological indicators [32]. Quantitative measurements of physiological and behavioral stress responses in fish can be made in the future using a combination of biosensors, triaxial acceleration sensors, and computer vision technology to comprehensively analyze the stress state of fish. In addition, while our simulation system successfully reproduced the key environmental stressor of shallow water depth, it cannot fully capture other environmental factors, such as temperature fluctuations, variations in dissolved oxygen levels, and bird predation, in actual paddy fields. Future work will involve deploying biosensors alongside multiparameter environmental monitoring (e.g., temperature and dissolved oxygen) to validate the evolutionary fitness of the Qingtian paddy field carp in real paddy fields.

### 4.2. Oxidative Damage Recovery: An Evolutionary Signature

Glucose alone cannot fully explain the complexity of stress-related behaviors. Therefore, we integrated antioxidant and biochemical indices from multiple tissues to provide a more comprehensive understanding of stress adaptation. In this study, antioxidant capacity was measured in the hepatopancreas, gill tissues, and serum to capture a comprehensive view of the stress response. These three tissues are functionally interconnected: the gills serve as the primary interface with the external environment, directly experiencing external stressors; the hepatopancreas is the central metabolic and detoxification organ, synthesizing antioxidant enzymes and regulating energy supply; and serum integrates signals from multiple tissues, reflecting the systemic physiological status. By analyzing these three levels simultaneously, we provide a holistic understanding of carp adaptation to acute shallow-water conditions [33,34,35].

Under acute shallow-water stress, Qingtian paddy field carp and Xingguo red carp significantly differed in oxidative stress responses. As a reliable indicator of oxidative stress and inflammatory responses, MDA reduction is considered an effective strategy for mitigating oxidative damage [36,37]. Under short-term stress, MDA levels in hepatopancreas and gill tissues showed significant elevation in both fish, indicating the rapid activation of the membrane lipid peroxidation cascade by oxidative stress [38]. However, during long-term stress, Qingtian paddy field carp showed a significant reduction in MDA levels, while Xingguo red carp failed to restore MDA, indicating that Qingtian paddy field carp may activate repair mechanisms, such as antioxidant enzyme systems or membrane lipid remodeling, similar to the adaptive regulation observed in Nile tilapia (*Oreochromis niloticus*) under salinity stress [39]. In contrast, Xingguo red carp appeared to have limited capacity for repairing oxidative damage.

Further analysis of catalase (CAT) regulatory patterns revealed that the CAT activity in the hepatopancreas of Qingtian paddy field carp remained stable throughout stress exposure, with gill tissue activity recovering to control levels after long-term stress. This ability to maintain homeostasis may stem from a unique advantage in CAT isoform expression, which enables sustained decomposition of H_2_O_2_ and thereby prevents the accumulation of hydroxyl radicals [40]. This mechanism is consistent with the CAT stability characteristics reported in Pseudosciaena crocea (*Larimichthys crocea*) under salinity stress [41]. In contrast, Xingguo red carp showed significant hepatopancreatic CAT activity reduction after short-term stress, indicating impaired secondary antioxidant defense efficiency [42].

In addition, superoxide dismutase (SOD) activity in both fish species showed no significant changes in hepatopancreas and gill tissues, suggesting conservative SOD responses to acute stress, with stability potentially relying on synergistic effects of secondary antioxidant systems like glutathione metabolism [43]. Regarding glutathione (GSH) and total antioxidant capacity (T-AOC), Qingtian paddy field carp maintained stable gill GSH activity, whereas Xingguo red carp exhibited significant GSH elevation in prolonged stress groups, reflecting compensatory upregulation to counteract persistent oxidative damage, analogous to the response pattern of gibel carp (*Carassius auratus gibelio*) under heavy metal stress [44]. T-AOC initially rose in both species, but only Qingtian paddy field carp achieved dynamic equilibrium via CAT recovery and MDA degradation; the T-AOC of Xingguo red carp declined under continued stress, evidencing insufficient repair. Lactate dehydrogenase (LDH), a key enzyme in anaerobic metabolism, typically shows elevated activity, reflecting enhanced glycolysis under hypoxia [45]. Both species exhibited significantly increased LDH activity under the long-term stress group in hepatopancreas tissues, whereas gill tissues showed reduced LDH activity under long-term stress. As the primary organ directly exposed to stressors, the gills might downregulate ATP demand (e.g., through inhibition of Na+/K+-ATPase activity) to conserve energy and reduce oxygen consumption under environmental stress, while the hepatopancreas, as the metabolic hub, maintains glycolytic energy supply.

Notably, antioxidant enzyme activities in Qingtian paddy field carp did not exhibit significant changes under shallow-water stress, suggesting a relatively stable oxidative state. However, since high-throughput sequencing was not performed in this study, the underlying molecular mechanisms remain unclear. To address this limitation, future research will involve integrated transcriptomic and metabolomic analyses to elucidate the mechanistic basis of stress responses in conjunction with enzymatic activity profiles.

### 4.3. Metabolic Flexibility: Energy Strategy for Fluctuating Environments

Under acute shallow-water-level stress, the blood glucose (GLU) metabolism of Qingtian paddy field carp and Xingguo red carp exhibited significant differences. In the short-term stress group, serum GLU content significantly increased in both species, aligning with the general pattern of stress-induced hyperglycemia in fish, indicating the activation of the HPI axis to promote gluconeogenesis for meeting acute energy demands [46,47]. However, under long-term stress, Qingtian paddy field carp showed a significant GLU reduction while Xingguo red carp failed to recover, suggesting that the former might achieve blood glucose homeostasis through enhanced glucose transporter (GLUT) activity, reflecting its metabolic plasticity advantage [48,49]. The gradual increase in TC and TG levels under prolonged shallow-water stress suggests that Qingtian paddy field carp may activate lipid metabolism pathways to meet energy demands and maintain physiological homeostasis. This response is likely mediated by stress-induced hormonal changes and metabolic reprogramming, which promote lipid mobilization and circulation [50]. A similar metabolic shift has been observed in Nile tilapia (*Oreochromis niloticus*), in which carbohydrates serve as the primary energy source during acute hypoxia, whereas lipids become predominant under prolonged hypoxic conditions [51]. This demonstrates that Qingtian paddy field carp can rapidly adjust energy allocation to mitigate persistent stress-induced depletion of energy reserves, and that this lipid-based metabolic strategy may reflect an evolutionary adaptation to periodic fluctuations in food availability within paddy field habitats [7].

Serum ALT and AST activities, as sensitive biomarkers of hepatocyte damage, typically reflect increased cell membrane permeability or parenchymal hepatic injury when elevated [52]. Both species showed significant increases in serum ALT and AST activities in short-term stress groups. However, Qingtian paddy field carp demonstrated a notable decrease in ALT activity in prolonged stress groups, whereas Xingguo red carp displayed no recovery. Furthermore, the absence of significant fluctuations in serum albumin (ALB) activity in both species confirms the preservation of fundamental hepatic synthetic functions [53].

While our biochemical analysis revealed clear differences in antioxidant regulation among the hepatopancreas, the gills, and serum, we recognize that such indices provide limited insight into the underlying molecular mechanisms. Previous transcriptomic and metabolomic studies in Qingtian paddy field carp have demonstrated the upregulation of HSP-related genes, the modulation of glutamine/glutamate metabolism, and enhanced lipid mobilization under paddy field stress conditions [7,9,22,24,43]. These findings are consistent with our biochemical results and suggest that Qingtian paddy field carp possess an evolved regulatory network enabling efficient oxidative repair and metabolic adaptation. Future studies will integrate biosensor-based real-time monitoring with high-throughput omics approaches to uncover the molecular basis of stress tolerance more comprehensively.

## 5. Conclusions

In this study, we combined the superiority of continuous biosensing technology in dynamic stress physiological studies and the comprehensiveness of biochemical analytical techniques to investigate the changes in physiological indices of two carp species under shallow-water stress. Qingtian paddy field carp are more effective at withstanding shallow-water stress than Xingguo red carp. They maintain stable glucose homeostasis even when the water depth is reduced to an extreme level, recover antioxidant balance after prolonged exposure, and shift to lipid-based metabolism to sustain the energy supply. In contrast, Xingguo red carp exhibit rapid glucose elevation, persistent oxidative damage, and limited metabolic flexibility. As a result, Qingtian paddy field carp exhibit more robust adaptive mechanisms, shaped by long-term exposure to shallow-water paddy field environments. These findings underscore the comprehensive stress-resistance traits of Qingtian paddy field carp and provide a theoretical foundation for identifying stress-resistance molecular markers for selective breeding, while also validating the sustainability of the GIAHS Qingtian rice–fish co-culture system and offering a new framework for predicting species vulnerability to habitat changes under climate warming.

## Data Availability

The data supporting the findings of this study are available from the corresponding author, upon reasonable request.

## Abbreviations

The following abbreviations are used in this manuscript:

FAO	Food and Agriculture Organization
GIAHS	Globally Important Agricultural Heritage Systems
HPI	Hypothalamic–pituitary–interrenal
HSPs	Heat shock proteins
AMPK	AMP-activated protein kinase
GOx	Glucose oxidase
BSA	Bovine serum albumin
PBS	Phosphate-buffer solution
AISF	Abdominal interstitial fluid
SOD	Superoxide dismutase
MDA	Malondialdehyde
LDH	Lactate dehydrogenase
CAT	Catalase
GSH	Reduced glutathione
T-AOC	Total antioxidant capacity
TP	Total protein
GLU	Glucose
ALB	Albumin
ALT	Alanine aminotransferase
AST	Aspartate aminotransferase
TG	Triglyceride
TC	Total cholesterol
ANOVA	One-way analysis of variance
SEM	Standard error of the mean
QC	Qingtian paddy field carp control group
QS	Qingtian paddy field carp short-term stress group
QL	Qingtian paddy field carp long-term stress group
XC	Xingguo red carp control group
XS	Xingguo red carp short-term stress group
XL	Xingguo red carp long-term stress group

## Appendix A

**Table A1:** Information on the commercially available assay kits applied in this study.

Biochemical Parameter	Assay Kit Name	Coefficient of Variation	Detection Ranges
SOD	Superoxide Dismutase (SOD) typed assay kit (Hydroxylamine method)	1.7%	5–122.1 U/mL
MDA	Malondialdehyde (MDA) assay kit (TBA method)	2.3%	0–113 nmol/mL
LDH	Lactate dehydrogenase assay kit	1.5%	9.0–5000 U/L
CAT	Catalase (CAT) assay kit (Visible light)	1.7%	0.2–24.8 U/mL
GSH	Glutathione Peroxidase (GSH-PX) assay kit	3.1%	20–330 U/mL
T-AOC	Total antioxidant capacity assay kit	3.2%	0.2–55.2 U/mL
ALT	Alanine Aminotransferase Test Kit (IFCC Method)	3.5%	4–1000 U/L
AST	Aspartate Aminotransferase Test Kit (IFCC Method)	3.5%	4–800 U/L
ALB	Albumin Kit (BCG Method)	2.5%	3–60 g/L
TC	Total Cholesterol Determination Kit (COD—PAP Method)	3.0%	0.1–20.0 mmol/L
TG	Triglycerides Kit (GPO-POD Method)	3.0%	0.1–12.5 mmol/L
GLU	Glucose Assay Kit (Glucose Oxidase Method)	3.0%	0.3–25 mmol/L
